# Somatic *LKB1* Mutations Promote Cervical Cancer Progression

**DOI:** 10.1371/journal.pone.0005137

**Published:** 2009-04-02

**Authors:** Shana N. Wingo, Teresa D. Gallardo, Esra A. Akbay, Mei-Chi Liang, Cristina M. Contreras, Todd Boren, Takeshi Shimamura, David S. Miller, Norman E. Sharpless, Nabeel Bardeesy, David J. Kwiatkowski, John O. Schorge, Kwok-Kin Wong, Diego H. Castrillon

**Affiliations:** 1 Division of Gynecologic Oncology, UT Southwestern Medical Center, Dallas, Texas, United States of America; 2 Department of Pathology, UT Southwestern and Simmons Comprehensive Cancer Center, Dallas, Texas, United States of America; 3 Department of Medical Oncology, Dana Farber Cancer Institute, Boston, Massachusetts, United States of America; 4 Departments of Medicine and Genetics, Lineberger Comprehensive Cancer Center, University of North Carolina School of Medicine, Chapel Hill, North Carolina, United States of America; 5 Massachusetts General Hospital Cancer Center, Boston, Massachusetts, United States of America; 6 Department of Medicine, Brigham and Women's Hospital, Boston, Massachusetts, United States of America; 7 Division of Gynecologic Oncology, Massachusetts General Hospital, Boston, Massachusetts, United States of America; Health Canada, Canada

## Abstract

Human Papilloma Virus (HPV) is the etiologic agent for cervical cancer. Yet, infection with HPV is not sufficient to cause cervical cancer, because most infected women develop transient epithelial dysplasias that spontaneously regress. Progression to invasive cancer has been attributed to diverse host factors such as immune or hormonal status, as no recurrent genetic alterations have been identified in cervical cancers. Thus, the pressing question as to the biological basis of cervical cancer progression has remained unresolved, hampering the development of novel therapies and prognostic tests. Here we show that at least 20% of cervical cancers harbor somatically-acquired mutations in the *LKB1* tumor suppressor. Approximately one-half of tumors with mutations harbored single nucleotide substitutions or microdeletions identifiable by exon sequencing, while the other half harbored larger monoallelic or biallelic deletions detectable by multiplex ligation probe amplification (MLPA). Biallelic mutations were identified in most cervical cancer cell lines; HeLa, the first human cell line, harbors a homozygous 25 kb deletion that occurred *in vivo*. *LKB1* inactivation in primary tumors was associated with accelerated disease progression. Median survival was only 13 months for patients with *LKB1*-deficient tumors, but >100 months for patients with *LKB1*-wild type tumors (*P* = 0.015, log rank test; hazard ratio = 0.25, 95% CI = 0.083 to 0.77). *LKB1* is thus a major cervical tumor suppressor, demonstrating that acquired genetic alterations drive progression of HPV-induced dysplasias to invasive, lethal cancers. Furthermore, *LKB1* status can be exploited clinically to predict disease recurrence.

## Introduction

Cervical cancer is among the most common cancers worldwide, with over 500,000 new cases and 250,000 deaths each year. In the developing world, cervical cancer is the leading cause of cancer deaths in women [1]. Infection of cervical epithelial cells with a transmissible agent—the Human Papilloma Virus (HPV)—is necessary for the development of cervical cancer, as HPV DNA sequences are detectable in >99% of cervical tumors [2], [3]. Infection with “high-risk” HPV subtypes initiates tumor progression by abrogating cell cycle control and apoptosis checkpoints through the viral oncoproteins E6 and E7, which inactivate the p53 and RB tumor suppressor pathways respectively [2]. This leads to the formation of noninvasive (*in situ*) cervical dysplasias known as High-grade Squamous Intraepithelial Lesions, or HSILs) [3], [4], [5]. However, these HPV-induced dysplasias are asymptomatic and most regress, demonstrating that HPV is not sufficient to result in cervical cancer [2]. The progression of cervical dysplasias to invasive, lethal cervical cancers has been attributed to diverse factors such as immune, hormonal, and nutritional status, or co-infection with other sexually-transmitted agents, but supporting data have been equivocal [2]. Insertional mutagenesis by HPV is another proposed tumor-promoting mechanism, but recent studies have not supported this hypothesis [6]. No common, recurring genetic alterations that cooperate with HPV to promote cervical cancer progression have been identified since Harald Zur Hausen first identified HPV as the causal transmissible agent of cervical cancer over thirty years ago [7]. Thus, the pressing question as to the biological basis of cervical cancer progression has remained unresolved.

Germline mutations in the *LKB1* tumor suppressor gene (a.k.a. *STK11*) result in Peutz-Jeghers Syndrome (PJS), a hereditary condition characterized by benign gastrointestinal polyps and an elevated (>15×) risk of malignant epithelial cancers at various anatomic sites [8]. The *LKB1* gene was recently shown to undergo somatic mutation in >30% of non-small cell lung cancers [9], [10], suggesting that *LKB1* may play a broad tumor suppressor role. This, combined with our recent findings that *Lkb1* inactivation in mouse uterus or epidermis promotes aggressive endometrial and squamous cell carcinomas [11], [12] prompted us to explore the role of *LKB1* in cervical cancer progression.

## Results

### Somatically-Acquired *LKB1* Mutations are Common in Cervical Cancer Across Histologic Subtypes

**Figure 1:**
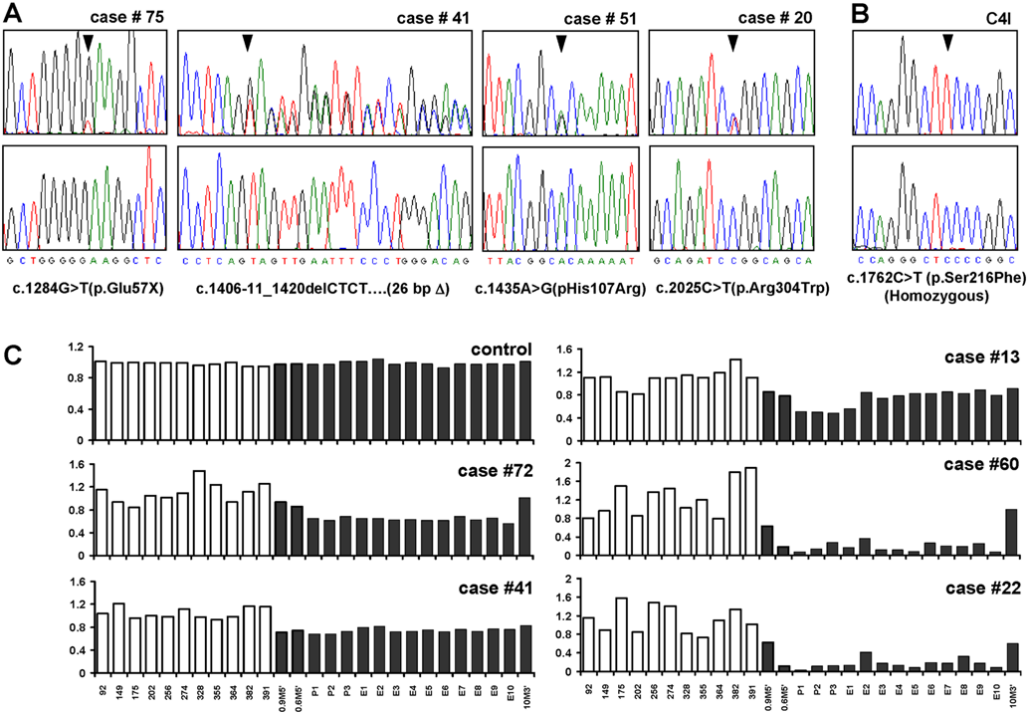
Somatic mutations and deletions of *LKB1* in cervical tumors. (A) Representative chromatograms of primary tumors. (B) C4I cell line. Lower panels, control DNA samples from each patient (for C4I, human peripheral leukocyte DNA). Wild-type sequences are shown below. Chromatograms represent forward strand except case #41 where reverse complement is shown to more clearly illustrate the deletion. Mutations are heterozygous except where indicated. (C) *LKB1* deletions in primary cervical tumors by MLPA. Bars = relative signal intensity per probe. Sixteen probes (black) correspond to *LKB1* locus on chromosome 19. Probe identifiers shown below. Probes 0.9M5′ and 0.6M5′ are ∼900 and 600 kb 5′ of locus (telomeric), while 10M3′ is ∼10000 kb 3′ (centromeric); remaining 13 probes correspond to *LKB1* noncoding/coding exons. White bars correspond to randomly selected probes from other chromosomes.

Sequencing of the *LKB1* gene in primary cervical tumors identified somatically-acquired (non-germline) mutations in 8/86 (9%) samples (Table 1, Table S1, Figure 1A). In addition to other findings presented below, several observations argue that these mutations are as a group inactivating, *bona fide* cancer-causing mutations. First, 4/8 tumors harbored nonsense mutations, deletions, or insertions resulting in frameshift and premature termination. The remaining four tumors harbored kinase domain mutations in residues conserved in vertebrate species, and two of these tumors harbored a known PJS mutation (p.Arg304Trp) that abrogates LKB1 kinase activity [13], [14], [15]. Lastly, only 1/9 coding variants were of germline origin, vs. 7/7 noncoding variants, a difference that is statistically significant (p = 0.0014, Fisher's Exact Test) particularly since the single germline coding variant *c.2077C>G* (p.Phe354Leu) is a known non-pathological neutral variant present in normal individuals [16]. Sequencing also identified a homozygous *LKB1* kinase domain mutation in the cervical cancer cell line C4I (Figure 1B, Table S1).

**Table 1:** Characteristics of 86 Patients in the Study Population.

**Age—yr**	
Median	46
Mean	47.1
Range	24–88
**Histology—number (%)**	
Adenocarcinoma	22 (26)
Adenosquamous	7 (8)
Squamous	57 (66)
**Stage—number (%)**	
I	25 (29)
II	36 (42)
III	15 (17)
IV	7 (8)
Unknown	3 (4)
**Grade—number (%)**	
1	3 (4)
2	57 (66)
3	18 (21)
Ungraded	8 (9)

**Figure 2:**
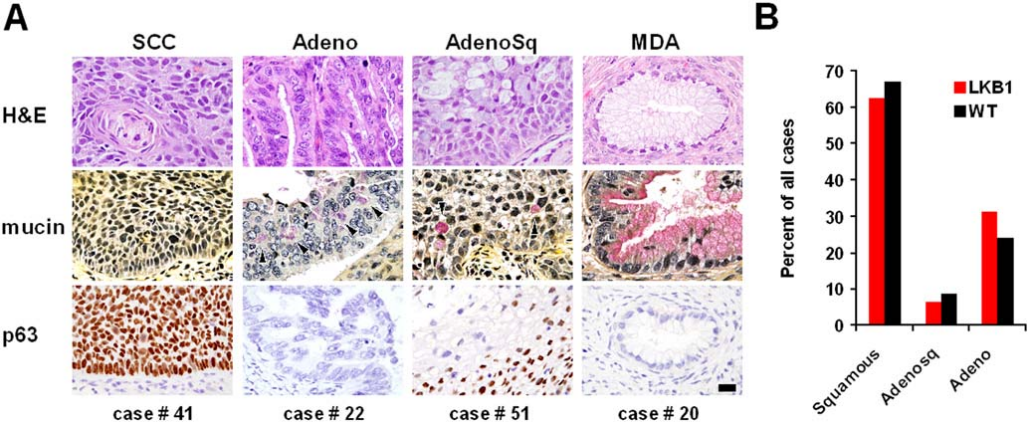
*LKB1* mutations occur in each of the principal histologic subtypes of cervical cancer. (A) Histology of representative cases with *LKB1* mutations (SCC = squamous cell carcinoma; Adeno = adenocarcinoma; AdenoSq = adenosquamous carcinoma; MDA = minimal deviation adenocarcinoma). Scale bar = 20 microns. (B) Relative distribution of the three principal histologic subtypes among *LKB1*-mutant (red) vs. *LKB1*-wild type (black) cases (totals = 100%) shows that the histologic spectrum is virtually identical in *LKB1* null vs. wild-type tumors.

Although one small (26 bp) *LKB1* deletion representing a complete loss-of-function was identified by sequencing (Table S1, Figure 1A), larger deletions would have been missed. To systematically screen for deletions, multiplex ligation-dependent probe amplification (MLPA) for all 10 *LKB1* exons and three flanking probes was employed. Whereas normal human DNA control samples (n = 3) consistently showed equivalent signal strengths for all probes, MLPA identified distinct *LKB1* deletions in 10/86 tumors (Table S1, Figure 1C). In five tumors, deletions appeared to be homozygous because signals from contiguous probes were reduced by >50% (Figure 1C, cases 60 and 22); residual signal in these cases likely reflects stromal contamination. Of tumors with apparent heterozygous deletions, one also contained a significant mutation identified by sequencing (Case 41, which harbors a 26 bp deletion, Table S1). These findings are consistent with human and mouse studies showing that although loss of the second allele can accelerate tumor progression, mutation of a single *LKB1* allele is by itself tumorigenic (i.e. *LKB1* can be haploinsufficient) [9], [17]. However, it is also possible that some mutations went undetected, or that stromal contamination led to an underestimation of homozygosity. In summary, biologically-significant *LKB1* mutations including deletions characterize at least 20% (17/86) of invasive cervical cancers. Only one case in our study (case 20, which harbored a somatically-acquired *LKB1* mutation) was diagnosed as a minimal-deviation adenocarcinoma (MDA), a rare, extremely well-differentiated variant of cervical adenocarcinoma associated with Peutz-Jeghers Syndrome [18]. Thus, *LKB1* mutations in cervical cancers were not limited to this rare histologic subtype but were present across the principal histologic subtypes of cervical cancer—adenocarcinoma, squamous cell carcinoma, and adenosquamous carcinoma (Figure 2, Table S1) [4].

### Deletions of *LKB1* Characterize Most Cervical Cancer Cell Lines

**Figure 3:**
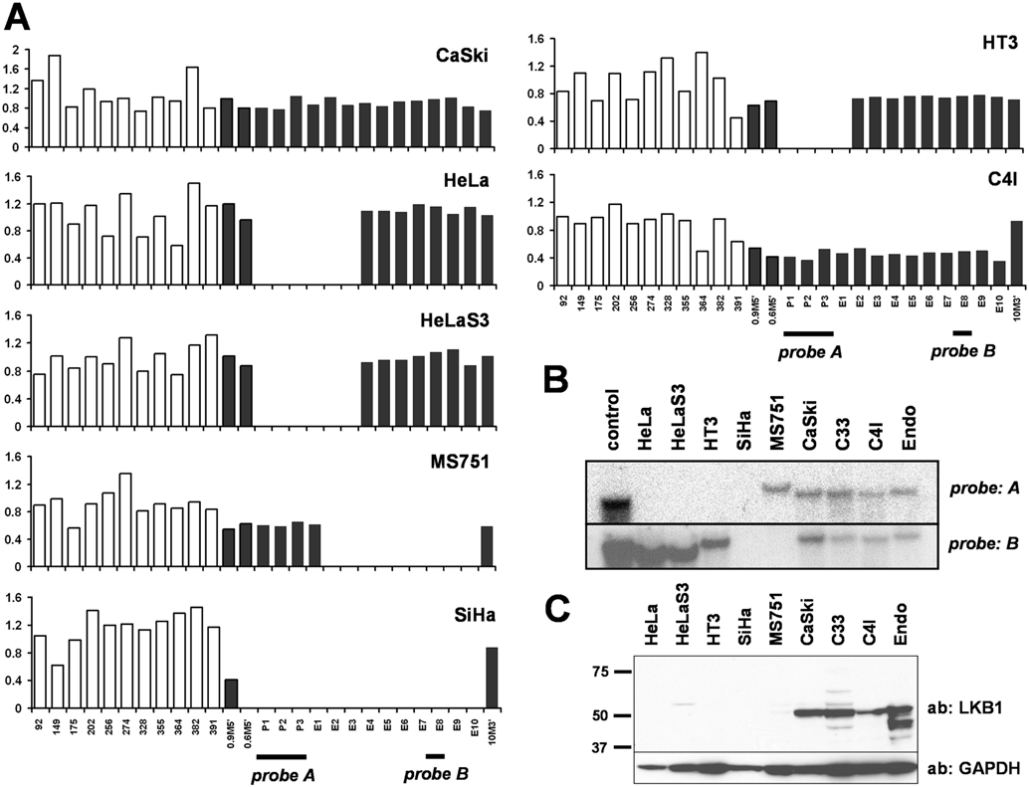
Homozygous *LKB1* deletions occur in majority of cervical cancer cell lines. (A) MLPA; see Figure 1 for probe details. HeLa, MS751, SiHa, and HT3 (and HeLa subclone HeLaS3) contain distinctive homozygous deletions. C4I harbors a monoallelic deletion, as evidenced by contiguous probes with half-intensity signals. (B) Southern analysis of control DNA, above cell lines, C33 (no deletion by MLPA) and HPV16/E6E7-immortalized endocervical cells from a normal patient (Endo = End1/E6E7; ATCC #CRL-2615). (C) Western analysis. LKB1 protein is undetectable in lines harboring homozygous deletions and decreased by ∼50% in C4I, consistent with monoallelic loss.

Primary tumors are clonally diverse and contain variable numbers of cells such as fibroblasts and lymphocytes that can obscure these and other analyses. To overcome this limitation and gain further insights into the role of *LKB1* in cervical cancer, a panel of seven cervical cancer cell lines (HeLa, HT3, SiHa, MS751, CaSki, C33a, and C4I) was analyzed. Strikingly, five of these seven cell lines harbored *LKB1* deletions, as did the HeLa derivative HeLaS3 (Figure 3A). Only the CaSki (Figure 3A) and C33a (not shown) cell lines did not exhibit *LKB1* deletions. The majority (4/7) harbored homozygous deletions, while the one cell line with a heterozygous deletion (C4I) also harbored a point mutation (Figure 1B, Table S1) rationalizing the loss of heterozygosity for this mutation in C4I. Southern analysis confirmed loss of *LKB1* sequences (Figure 3B) and as expected, LKB1 protein was undetectable in the cell lines harboring homozygous deletions (Figure 3C). This frequent homozygosity of *LKB1* mutations in cell lines further underscores the pathogenetic significance of *LKB1* loss in cervical cancer. Although the number of lines available and hence analyzed was small, it is notable that the majority of cervical cancer cell lines harbored definitive bialleleic *LKB1* mutations. It is also possible that the establishment of primary cervical tumor cultures is biased towards *LKB1*-deficient tumors.

The absence of LKB1 protein in HeLa and HeLaS3 has been previously noted [19]. The *LKB1* gene is associated with a prominent CpG island, and the lack of LKB1 protein and mRNA in HeLa cells had been previously attributed to promoter hypermethylation [20]. However, we found no evidence of *LKB1* hypermethylation by methylation-specific PCR in any cell line or primary tumor samples. To the contrary, our data show that HeLa and other cervical cancer cell lines do not express *LKB1* because of homozygous deletions, rather than as a result of epigenetic silencing. Consistent with prior studies carried out in HeLa [19] and other cell lines [9], enforced expression of wild-type LKB1 led to cell cycle arrest and growth inhibition, demonstrating that *LKB1* loss in these cell lines is functionally significant (data not shown).

**Figure 4:**
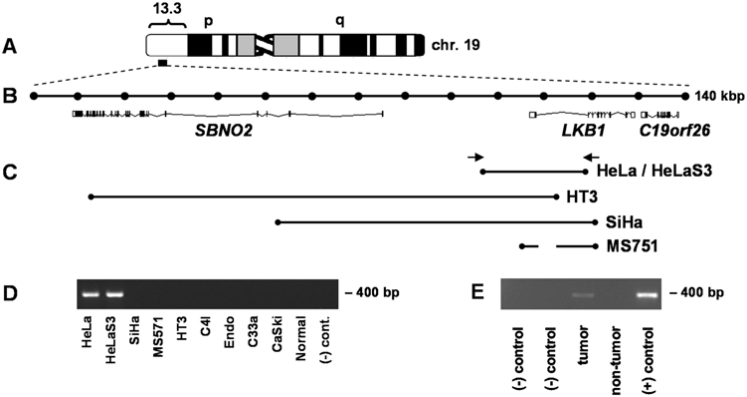
*LKB1* deletion breakpoints in cervical cancer cell lines. (A) *LKB1* locus (chromosome 19p13.3). (B) 140 kb region (Ensembl50; 1050000–1190000) spanning *LKB1* and immediately flanking loci; intervals = 10 kb. (C) Deletion breakpoints for cell lines harboring homozygous deletions. In MS751, deleted sequences are discontinuous as shown, consistent with a more complex rearrangement. Arrows = PCR primers for HeLa specific PCR (400 bp). (D) HeLa specific PCR (400 bp) confirms presence of deletion in HeLaS3. (E) HeLa intragenic *LKB1* deletion occurred *in vivo*. Archival blocks were cored for DNA preparation. Lanes are as follows: (−) control = no template control; tumor = metastatic adrenal deposit; non-tumor = normal adrenal (same tissue block); (+) control = HeLa cell line DNA.

To define deletion breakpoints, PCR reactions producing small amplicons (100–200 bp) were designed to span the locus (Figure 4A, B). Scoring (+/−) amplification of each product followed by additional PCR reactions for progressively narrower intervals based on results of the initial scan permitted us to map breakpoints to within a few kb (Figure 4C). Each of the four cell lines harbored a distinct deletion (∼20–110 kb) removing portions of *LKB1* and at most one other flanking locus (*SBNO*2).

### Molecular Cloning of the HeLa *LKB1* Deletion Junction and *In Vivo* Origin of the Deletion

To clone across the HeLa junction, primers flanking the 5′ and 3′ breakpoints mapped by the above PCR scanning strategy were designed to amplify a specific breakpoint product. A 2.8 kb junctional fragment was cloned and sequenced, confirming the presence of a deletion (24,662 bp, Figure S1, Figure 4C). Both breakpoints lie within Alu repeats (Figure S1), suggesting that inter-Alu homologous recombination produced the deletion. To create an efficient PCR assay for this deletion, primers immediately flanking the repetitive Alu sequences at each breakpoint were designed (400 bp) (Figure S1).

HeLaS3, a clonal derivative of HeLa first reported in 1955 [21], harbored the identical *LKB1* deletion (Figure 4D), establishing 1955 as a *terminus ante quem* for the origin of the deletion. However, it remained formally possible that the *LKB1* deletion arose *in vitro* following the establishment of HeLa from a primary cervical adenocarcinoma in 1951 [22]. To resolve this question, HeLa tumor DNA was isolated from an archival paraffin-embedded tissue block prepared in the course of the patient's autopsy at the Johns Hopkins Hospital in 1951. A 400 bp HeLa-specific PCR product was amplified from the tumor (Figure 4E), establishing that the *LKB1* deletion occurred *in vivo*.

**Figure 5:**
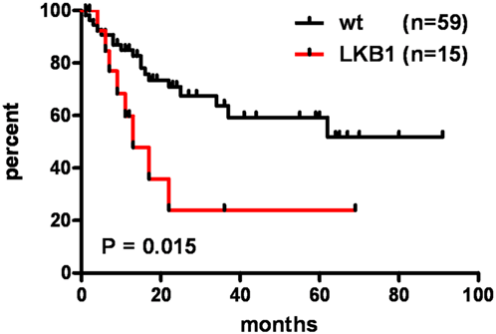
Progression-free survival of patients with *LKB1*-wild type vs. *LKB1*-mutant tumors. Kaplan-Meier curves show the percentage of patients with disease progression over time. The curves compare patients whose tumors were heterozygous or homozygous for mutations/deletions (*LKB1*) vs. patients with no mutations/deletions (wt). Patients with >1 follow-up visit were included in the analysis. P value per log-rank test.

The occurrence of the *LKB1* HeLa deletion while the patient was alive suggested that *LKB1* inactivation might have contributed to the notoriously aggressive growth of her tumor both *in vivo* and *in vitro* (see below and discussion). To explore the possibility that *LKB1* mutations influence disease course, progression-free survival curves were generated for patients whose tumors harbored *LKB1* mutations identified by sequencing or MLPA (heterozygous or homozygous) and patients whose tumors were wild-type for *LKB1*. Strikingly, the median survival was only 13 months for patients with *LKB1*-deficient tumors, but >100 months for patients with *LKB1*-wild type tumors (Figure 5) (P = 0.015, log-rank test; hazard ratio = 0.25, 95% CI = 0.083 to 0.77). *LKB1*-deficient tumors were not more advanced at the time of staging, e.g., 28% of *LKB1*-wild type tumors were initially Stage I (confined to the uterus), vs. 43% for *LKB1*-deficient tumors, nor of higher grade (i.e. more poorly-differentiated). Thus, *LKB1* mutations in cervical tumors confer a stage-for-stage increased risk of recurrence, suggesting that assays of *LKB1* status will be of clinical utility in identifying patients at increased risk for disease progression.

## Discussion

Our study is the first to demonstrate that recurrent genetic mutations occur in cervical cancer. An earlier study that examined this question did not identify *LKB1* mutations in sporadic cervical cancers, but only minimal deviation adenocarcinomas were analyzed, as the goal of the study was to ascertain *LKB1* mutation frequencies in gynecologic malignancies known to be associated with PJS. However, this earlier study, performed prior to the advent of MLPA, identified LOH of the *LKB1* 19p13.3 region in 3/8 cases, suggesting that *LKB1* deletions may have been present [23]. Another study where 26 cervical tumors were analyzed by single-strand conformational polymorphism analysis identified *LKB1* mutations in only one case [24]. However, this study also greatly underestimated the frequency of *LKB1* mutations in lung cancer, now known to occur in >20% of cases. The advent of MLPA now facilitates the definitive identification of *LKB1* deletions, which account for ∼50% of mutations in lung and cervical cancer (this and other studies) [9]. Another factor potentially obscuring prior sequence analyses is the unusually high GC content of *LKB1* exonic regions. In our experience, automated base call software overlooked a high percentage of mutations, necessitating direct inspection of chromatograms.

The discovery of a homozygous *LKB1* deletion in HeLa is noteworthy because of the historical significance of this cell line to biomedical research. HeLa was derived in 1951 from a cervical adenocarcinoma. As the first immortalized human cell line isolated and successfully perpetuated *in vitro*, HeLa greatly accelerated the progress of biomedical research in the second half of the 20^th^ century [25]. HeLa cells were unusual in growing so rapidly in culture, but the primary tumor was also aggressive. The primary tumor was confined to the cervix at the time of diagnosis, but it metastasized early and widely despite aggressive therapy including radiation treatment, leading to the patient's death just six months after the initial diagnosis [26], [27], [28]. Our results suggest that the homozygous deletion we have documented within *LKB1* contributed to this aggressive growth phenotype, rationalizing some of the unusual features of the HeLa primary tumor and cell line. Consistent with this idea, enforced expression of *LKB1* cDNA into HeLa and other HeLa-deficient cell lines induces growth arrest (unpublished data, see also [9], [19]).

This is also the first report that *LKB1* mutations confer a worse prognosis for a particular human cancer. However, this observations is in line with genetically-engineered mouse models of *LKB1* deficiency that have consistently found that Lkb1 loss promotes invasive/metastatic growth. In *K-ras* driven mouse models of lung cancer, *Lkb1* inactivation provided the strongest cooperation in terms of tumor latency and frequency of metastasis (as compared to classic tumor suppressors such as *p53* and *Ink4a/Arf*). It is also notable that *Lkb1* loss in the lung was associated with a broad histologic spectrum (squamous cell carcinomas to adenocarcinomas), recalling that *LKB1* inactivation characterizes cervical carcinomas of varied histologic subtypes [9]. Similarly, mice with targeted inactivation of *Lkb1* in endometrial epithelium develop highly invasive (yet paradoxically extremely well-differentiated) endometrial adenocarcinomas [11]. Taken together, these and other results [12], [29] argue that LKB1 suppresses invasion and metastasis and suggest that assays based on LKB1 may prove useful for prognostication in a variety of cancers. It will be of interest to determine if *LKB1* mutations are also useful prognostically in other cancers [9].

The fact that 99% of cervical cancers contain HPV DNA [2] as is the case for most of the cervical cancer cell lines in which we demonstrated homozygous *LKB1* deletions (e.g. HeLa harbors integrated HPV-18 sequences) [30] suggest that *LKB1* and HPV cooperate in some manner. HPV infection promotes the formation of *in situ* lesions, leading us to propose that additional mutations in genes including *LKB1* are required to convert *in situ* dysplasias to invasive carcinomas, an idea consistent with the diverse animal models discussed above. Further studies i.e. with genetically-engineered animal models will be required to understand the biological basis of the interaction between HPV and LKB1. The biological and biochemical basis of LKB1-mediated carcinogenesis remains to be fully elucidated. Misregulation of the AMPK-mTOR pathway likely contributes to LKB1's role as a tumor suppressor, but probably does not entirely account for its role in mediating invasion [31]. Nonetheless, misregulation of mTOR in LKB1-deficient tumors may present opportunities for targeted therapy (e.g. through the use of metformin or rapamycin analogs) in women whose cervical tumors have confirmed *LKB1* mutations/deletions, an idea that merits further investigation in the future.

The biological basis of the progression of HPV-induced precancers has been undefined. This study presents the first definitive evidence that recurring mutations in discrete host genes occur in invasive cervical cancer. Although other factors likely influence progression, this study demonstrates that a process likely to be stochastic—namely the acquisition of discrete genetic mutations—drives progression of cervical dysplasias to invasive lethal carcinomas and that these mutations have the potential to serve as useful prognosticators.

## Materials and Methods

### Ethics Statement

This study was conducted according to the principles expressed in the Declaration of Helsinki. The study was approved by the Institutional Review Board of the UT Southwestern and Johns Hopkins hospitals.

### Patient Samples and Cell Lines

The patients were a subgroup of patients diagnosed with primary cervical cancer at UT Southwestern University Hospitals between 2000–2007 and who provided informed consent. Histopathologic diagnoses were made per standard criteria [4]. Sufficient amounts of tissue from the primary tumor had to be available to permit the isolation of DNA for sequencing and MLPA; otherwise there were no exclusion criteria. For most patients, control DNA was prepared from blood; for a small number of patients for whom blood was unavailable, DNA was prepared from paraffin-embedded control tissues. Tumor stage was determined per FIGO criteria. Data for disease progression analysis per RECIST criteria were obtained from follow-up visits. We recommended that patients have follow-up visits every 3 months for 2 years, then every 6 months for 3 years, and annually thereafter. For progression-free survival, time from completion of primary therapy to recurrence or death was recorded. Data for patients without tumor recurrence were censored at the time of the last follow-up visit. For the HeLa tumor study, a paraffin block was obtained from the archives of the Johns Hopkins Hospital. Cell lines were purchased from the ATCC.

### DNA Sequencing

DNA was prepared using Qiagen genomic DNA columns. A two step “boost/nest” PCR strategy was employed where the boost reaction generated a larger fragment used as a template for the nest reaction. The nest products were bidirectionally sequenced on ABI 3730 XL sequencers with ABI Big Dye Terminator 3.1 chemistry. Base calling was performed with the Agent system (Paracel). Sequence tracings were visually inspected to confirm accurate variant detection by the base-calling software. PCR primer sequences/conditions are available upon request. Coding variants were confirmed on repeat PCR reactions to exclude PCR artifacts. Genbank NM_000455 was used as the reference cDNA for nucleotide positions.

### Multiplex Ligation Dependent Probe Amplification (MLPA) and Southern/Western Analysis

MLPA was performed as described [9]. For Southern analysis, 10 µg genomic DNA was *XbaI*-digested prior to gel electrophoresis, transferred to a membrane, and hybridized with radiolabelled-probes (1–2 kb) generated by PCR and confirmed by end-sequencing. The membrane was hybridized to probe A, subjected to autoradiography, stripped in boiling 0.1% SDS, and rehybridized to probe B. For Western analysis, protein extracts were prepared from adherent cells by homogenization in lysis buffer+protease inhibitors on ice, subjected to SDS-PAGE and immunoblotted with an LKB1 antibody (#3047, Cell Signalling Technology) per the manufacturer's recommendations.

### Tissue Staining and Immunohistochemistry

Paraffin block 5 µm sections were deparaffinized/ hydrated in an ethanol series. Slides were stained with H&E and mucicarmine per standard protocols. For immunohistochemistry, slides were subjected to antigen retrieval in boiling in 10 mM NaCitrate; p63 antibody (#MS1081, LabVision) was used at 1∶400 with the Immpress detection system (Vector).

### PCR Scanning to Define Deletions and HeLa-specific PCR

PCR primers (producing 100–200 bp amplicons) spanning the *LKB1* region were designed using Primer3. Primer pairs producing products of correct size when normal human DNA was used as template were used to map deletions by scoring presence/absence of amplification with cell line DNAs as template. Additional reactions to give products corresponding to progressively smaller intervals were designed as necessary. Primer sequences and PCR conditions are available upon request. Archival tissue block DNA was prepared as described [32]. Primer sequences for HeLa-specific PCR (400 bp) were For(5′-GGTTGCGATCAAGGCCCCGA-3′) and Rev(5′-GCCTGTGGATGCCACACATG-3′). PCR conditions were 95C×10′; 94C×30″, 58C×30″, 72C×30″ (38 cycles); 72C×7′.

### Statistical Analysis

For comparison of mutant frequencies, Fisher's (two-tailed) unpaired exact test was used. Survival curves were generated in GraphPad Prism5, with comparison of the curves performed with the Log-rank (Mantel-Cox) test. The hazard ratio and its confidence interval were computed using the Mantel-Haenszel method. P values less than 0.05 were considered to indicate statistical significance.

## Supporting Information

Figure S1 Cloning and characterization of *LKB1* intragenic deletion breakpoints in HeLa/HeLaS3. A 2.8 kb fragment spanning the deletion breakpoint was cloned from HeLa DNA by PCR as described in the text; 974 bp of this sequence are shown. Sequence in bold red letters corresponds to the Alu repeat in which the presumptive homologous recombination event resulting in the HeLa deletion occurred. Nucleotide polymorphisms in the two native Alu sequences (not shown) were consistent with a recombination event occurring within the 5 bp boxed sequence (i.e., the flanking G bases were polymorphic and informative). Sequence in blue corresponds to a unique sequence in the human genome (Ensembl 50 19:1145482 to 1145865). This sequence is ∼11 kb from the 5′ end of the *LKB1* gene (transcriptional start). Sequence in green (Ensembl 50 19:1170845 to 1171115) lies within *LKB1* intron 3–4. The arrows show the location of the primers designed for HeLa-specific PCR (400 bp amplicon). The genomic location of these primers on the genomic map is shown in Figure 4C.
(0.01 MB PDF)

Table S1 Complete list of LKB1 mutations detected by sequencing or MLPA.
(0.01 MB PDF)
