# Supplementary material for: Somatic LKB1 Mutations Promote Cervical Cancer Progression
Source: PLoS One. 2009 Apr 2;4(4):e5137. doi: 10.1371/journal.pone.0005137 (PMC2660434; doi:10.1371/journal.pone.0005137)
Supplement: Figure S1 — Cloning and characterization of LKB1 intragenic deletion breakpoints in HeLa/HeLaS3. A 2.8 kb fragment spanning the deletion breakpoint was cloned from HeLa DNA by PCR as described in the text; 974 bp of this sequence are shown. Sequence in bold red letters corresponds to the Alu repeat in which the presumptive homologous recombination event resulting in the HeLa deletion occurred. Nucleotide polymorphisms in the two native Alu sequences (not shown) were consistent with a recombination event occurring within the 5 bp boxed sequence (i.e., the flanking G bases were polymorphic and informative). Sequence in blue corresponds to a unique sequence in the human genome (Ensembl 50 19:1145482 to 1145865). This sequence is ∼11 kb from the 5′ end of the LKB1 gene (transcriptional start). Sequence in green (Ensembl 50 19:1170845 to 1171115) lies within LKB1 intron 3–4. The arrows show the location of the primers designed for HeLa-specific PCR (400 bp amplicon). The genomic location of these primers on the genomic map is shown in Figure 4C. (0.01 MB PDF) [file pone.0005137.s001.pdf]

ATCGGATTATTTGCAGCGGATCCTACACGTCCCCTCCTCCTGCCTCTTGCCCTGCGCTCCCGAAG
GGGCCTCCGGACCCGGAGGATCTCACCGCGGGGCGGGGTCCACGGCGAGGGGGCTCGGCCAAGCG
GGCAGCCTCGCGCACGCGCACTCAACCCGGACGCGGGCCTCTCCGCCGCAGCCCGCGCCCGCCAC
CTGGCGGCCACAGCGGTCCGCGCAGACCGCCGGCCCCTTTTCTATCCGCGACCGGATACCTGGCA
GGGGTCTAGGAAAGACCCCTCTGGCTGCTGCGGGGCCAGTGCGTGCGAGGCTCGACCCCACTCC
ATTGCGTGGGGGGGCTGAGCTAGGTCCGTGAAGCGGGGC*GGTTGCGATCAAGGCCCCGA*CTTTTT
TTTTTTTTTTTTTTTTTTTTTTTTTGAGACGGAGTCTCGCTCTGTCGCCCAGGCCGGACTGCGGA
CTGCAGTGGCGCCATCTCGGCGCACTGCAACCTCTACCTCCCGGGTTCAAGCGATTCTCCTGCCT
CAGCCTCCCGAGTAGCTGGGACTACAGGCGCCCGCCACCGCGCCCGGCTAATTTTTTGTATTTTT
AGTAGAGACGGGGTTTCACCGTGTTAGCCAGGATGGTCTCAAACTCCTGACCTCGTGATCCGCCT
GCCTCAGCCTCCCAAAGTGCTGGGATTACAGGCATGAGCTACCACGCCCGGCCTTGTAAAGGCCC
AAGTTTTTAAAAACAGTTTTGGGGTCCCC*CATGTGTGGCATCCACAGGC*AGGGCTGCTGCCAACC
TCCCGCCTCCATCTTTGCTGGGCCTGCTGCCTGAGGCCAGTGGCCTGCTTCCAGCCCATCGCTGG
CAGCCGCCTGCCCTGACCAGATCTCCTGGATGCAGGTCTGTGGCCTCAGAGTCAGGGCCCCTTGC
TGCTGCAGGACCACAGGGGCAGGGAGGGGCCTGCTGTTCCAGCAAGACTTTGGGGTGCAGCCGGC
(974 bp shown)

Figure S1
