# Supplementary material for: Somatic LKB1 Mutations Promote Cervical Cancer Progression
Source: PLoS One. 2009 Apr 2;4(4):e5137. doi: 10.1371/journal.pone.0005137 (PMC2660434; doi:10.1371/journal.pone.0005137)
Supplement: Table S1 — Complete list of LKB1 mutations detected by sequencing or MLPA. (0.01 MB PDF) [file pone.0005137.s002.pdf]

Table S1. Complete list of *LKB1* mutations detected by sequencing or MLPA*.

Primary Tumors

Case ID	Stage	Histology	Sequencing Mutation(s)	MLPA (Copy Number Alterations)	Comment
23	IIB	Adenosquamous	*c.1171C>A (p.Ser19X)*<br>*c.1132C>A (p.Pro6Gln)*		Nonsense + missense mutation
75	IIB	Squamous	*c.1284G>T (p.Glu57X)*	-	Nonsense mutation
41	IIIB	Squamous	*c.1406-11_1420delCTCTGTCCCAGGG AAATTCAACTACT*	Single copy loss 0.9M5'-10M3'	26 bp deletion intron/exon boundary and frameshift
51	IIB	Squamous	*c.1435A>G (pHis107Arg)*	-	Kinase domain, phylogenetically conserved
19	IB	Adenocarcinoma	*c.1994_1995insA (p.Pro294fs)*	-	Frameshift and premature termination
14	IIA	Adenocarcinoma	*c.2009C>A (p.Phe298Leu)* (homozygous)	-	Kinase domain, phylogenetically conserved
16	IB2	Adenocarcinoma	*c.2025C>T (p.Arg304Trp)*	-	Known kinase-dead PJS mutation
20	IB2	Adenocarcinoma (MDA)*	*c.2025C>T (p.Arg304Trp)*	-	Known kinase-dead PJS mutation
13	IIIB	Adenocarcinoma		Single copy loss P1-E1	
22	IB2	Adenocarcinoma		Two copy loss 0.6M5'-E10	
30	IB2	Squamous	-	Single copy loss P1-E1	
48	IIIB	Squamous	-	Single copy loss 0.9M5'-10M3'	
50	IIB	Squamous	-	Two copy copy loss 0.6M5'-E10	
60	IIB	Squamous	-	Two copy loss 0.6M5'-E10	
62	IIB	Squamous	-	Two copy loss P1-E10	
72	IB2	Squamous	-	One copy loss P1-E10	
85	IB1	Squamous	-	Two copy loss P1-E1, one copy loss 0.9M5'-E10	

Cell Lines

C4I	-	Squamous	*c.1762C>T (p.Ser216Phe)* (homozygous)	Single copy loss P1-E10
HeLa	-	Adenocarcinoma	-	Two copy loss P1-E3
HeLaS3	-	Adenocarcinoma	-	Two copy loss P1-E3
MS751	-	Squamous	-	Two copy loss E2-E10
SiHa	-	Squamous	-	Two copy loss 0.65M5’-E10
HT3	-	Squamous	-	Two copy loss P1-E1

*MDA = minimal deviation adenocarcinoma
